# The impact of COVID-19 on ophthalmology resident surgical experience: a retrospective cross-sectional analysis

**DOI:** 10.1186/s12909-022-03205-0

**Published:** 2022-03-04

**Authors:** Hursuong Vongsachang, Michael J. Fliotsos, Alice C. Lorch, Eric L. Singman, Fasika A. Woreta, Grant A. Justin

**Affiliations:** 1 Wilmer Eye Institute, Johns Hopkins University School of Medicine, 600 N. Wolfe St, MD 21287 Baltimore, USA; 2 Present address: Department of Ophthalmology, Yale School of Medicine, 333 Cedar St, CT 06510 New Haven, USA; 3 Department of Ophthalmology, Massachusetts Eye and Ear Infirmary, 243 Charles St, MA 02114 Boston, USA; 4 Department of Ophthalmology, University of Maryland School of Medicine, 655 W. Baltimore St, MD 21201 Baltimore, USA; 5 Department of Ophthalmology, Walter Reed National Military Medical Center, 4494 Palmer Rd. N, MD 20814 Bethesda, USA; 6 Department of Surgery, Uniformed Services University of the Health Sciences, Walter Reed, 4301 Jones Bridge Road, MD 20814 Bethesda, USA

**Keywords:** Graduate medical education, Clinical competence, Ophthalmologic surgical procedures, COVID-19

## Abstract

**Background:**

The coronavirus disease 2019 (COVID-19) pandemic caused significant disruption to in-office and surgical procedures in the field of ophthalmology. The magnitude of the impact of the pandemic on surgical training among ophthalmology residents is not known. This study aims to quantify changes in average case logs among United States (U.S.) ophthalmology residency graduates prior to and during the COVID-19 pandemic.

**Methods:**

Retrospective, cross-sectional analysis of aggregate, national data on case logs of U.S. ophthalmology residency graduates from 2012 to 2020. The yearly percent change in the average number of procedures performed in the Accreditation Council for Graduate Medical Education (ACGME) ophthalmology resident case logs were analyzed using linear regression on log-transformed dependent variables. The average percent change from 2019 to 2020 was compared to the average yearly percent change from 2012 to 2019 for procedures performed as the primary surgeon, and primary surgeon and surgical assistant (S + A), as well as procedures for which there are ACGME minimum graduating numbers.

**Results:**

Across all procedures and roles, average case logs in 2020 were lower than the averages in 2019. While average total cases logged as primary surgeon increased yearly by 3.2% (95% CI: 2.7, 3.8%, p < 0.001) from 2012 to 2019, total primary surgeon case logs decreased by 11.2% from 2019 to 2020. Cataract (-22.0%) and keratorefractive (-21.1%) surgery experienced the greatest percent decrease in average primary surgeon cases logged from 2019 to 2020. Average total cases logged as S + A experienced an average yearly increase by 1.2% (95% CI: 0.9,1.6%, p < 0.001) prior to 2020, but decreased by 9.6% from 2019 to 2020. For ACGME minimum requirements, similar changes were observed. Specifically, the average case logs in YAG, SLT, filtering (glaucoma), and intravitreal injections had been increasing significantly prior to 2020 (p < 0.05 for all) but decreased in 2020.

**Conclusions:**

These findings demonstrate the vulnerability of ophthalmology residency programs to a significant interruption in surgical volume. There is a critical need for development of competency-based, rather than volume-based, requirements to evaluate readiness for independent practice.

**Supplementary Information:**

The online version contains supplementary material available at 10.1186/s12909-022-03205-0.

## Background

The coronavirus disease 2019 (COVID-19) pandemic caused significant disruptions to in-office and surgical procedures in ophthalmology. Following guidance urging provision of only urgent or emergent care from March to July 2020 in the United States (U.S.), total encounters in ophthalmology fell nearly 80% [1–3]. Residency programs had to consider methods to supplement resident learning during this time, especially considering that these shutdowns occurred in the months prior to residency graduation in June 2020 [4–6]. Residency is a crucial time for residents to gain surgical skills in preparation for fellowship and/or career as a practicing ophthalmologist, primarily through guided exposure to diverse cases performed in the operating room. As the impact of the pandemic on ophthalmic surgical training nationally has not been demonstrated, this study aimed to quantify changes in surgical training among ophthalmology residents in the U.S. prior to and during the pandemic.

## Methods

We accessed aggregate data on average case logs performed as the primary surgeon, and both primary surgeon and surgical assistant (S + A) roles among graduating ophthalmology residents nationwide from 2012 to 2020, published by the Accreditation Council for Graduate Medical Education (ACGME) [7]. Graduating residents are those in the final year of their four-year ophthalmology residency. Residents record cases performed as either the primary surgeon or surgical assistant into an online ACGME case log system. Each academic year, the ACGME produces anonymized, aggregated datasets with descriptive statistics on cases logged for each procedure across all residents nationally. We downloaded spreadsheets containing this publicly available data from the ACGME website by year. S + A data were included in this study to capture the operative experience of some procedures, such as vitreoretinal and refractive surgeries, that are primarily performed in the surgical assistant role. Cases logged for procedures with minimum ACGME requirements were also included and analyzed separately for more granular detail on specific procedures required for graduation.

For 2012–2019 data, the yearly percent change in the average cases performed by graduating ophthalmology residents per procedure was analyzed using linear regression models on log-transformed response variables with robust variance. This analysis was conducted for procedures performed as surgeon, S + A, and those designated as minimum ACGME graduation requirements. For each procedure, the average yearly percent change from 2012 to 2019 was compared to the percent change in average case logs between 2019 and 2020. Analyses were conducted on Stata SE/15.1 (StataCorp, College Station, Texas) with statistical significance set at *p* < 0.05. This study abides by the tenets of the Declaration of Helsinki and received exemption from the Johns Hopkins Institutional Review Board.

## Results

**Fig. 1:**
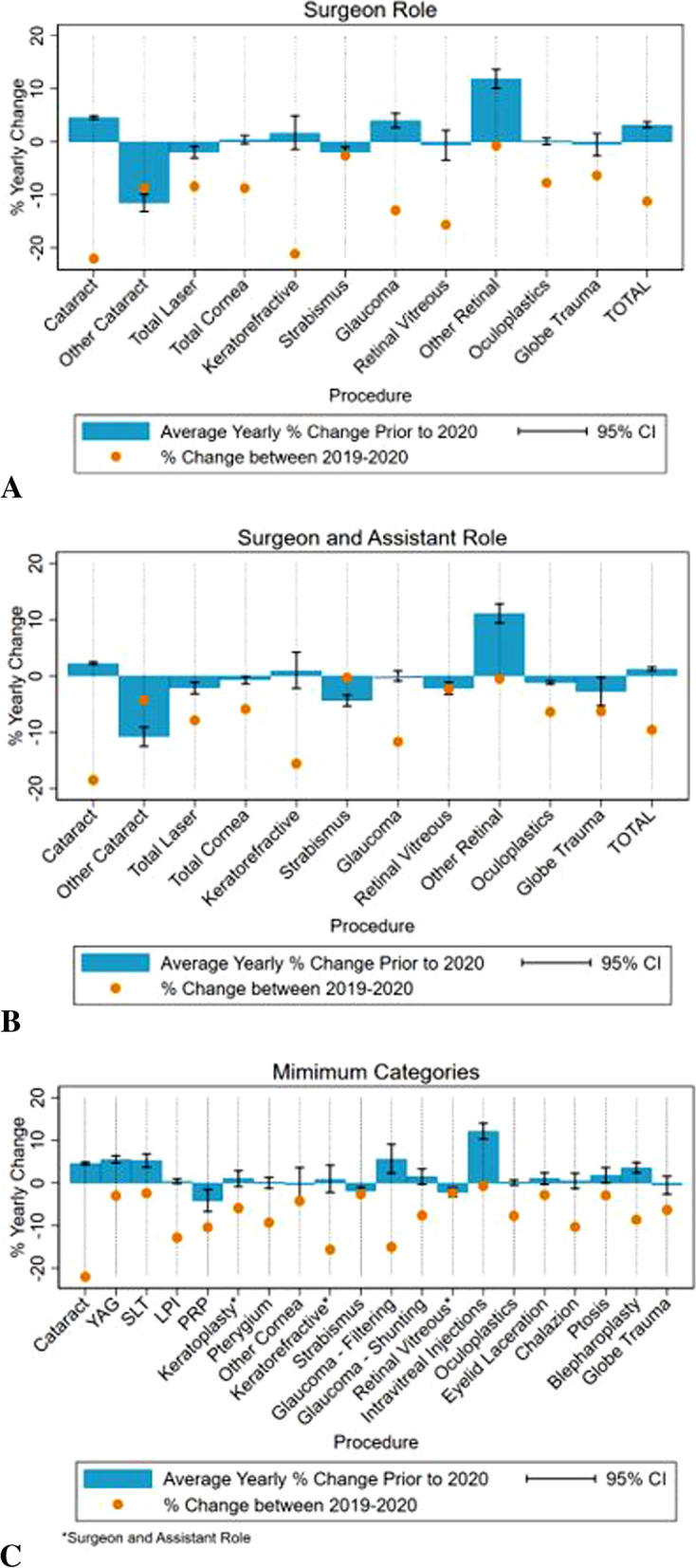
Comparison of the percent change in average case logs among U.S. ophthalmology residency graduates from 2019–2020 to the average yearly percent change from 2012–2019 for cases logged as **A** primary surgeon, **B** surgeon and assistant, and **C** procedures listed as minimum graduation requirements

Across all procedures and roles, average case logs in 2020 were lower than the averages in 2019. While average total cases logged as primary surgeon increased yearly by 3.2% (95% CI: 2.7, 3.8%, p < 0.001) from 2012 to 2019 (Table 1), total primary surgeon case logs decreased by 11.2% from 2019 to 2020. In the primary surgeon category, cataract, keratorefractive, and retina vitreous surgery experienced the greatest percent decreases in average cases logged from 2019 to 2020, at -22%, -21.1%, and − 15.6%, respectively (Fig. 1 A). Average primary surgeon cases in cataracts, glaucoma, and other retinal surgeries were generally increasing prior to 2020 (*p* < 0.001 for all) but declined in 2020. While average total laser cases were decreasing prior to 2020 (-1.9%, 95% CI: -3.0, -0.8%, *p* = 0.006), the decline was more pronounced in 2019–2020 at -8.4%. Cases logged as S + A were evaluated as well, of which the results are included in Fig. 1B and Table S1.

**Table 1:** Average Logged Cases across Graduating Residents by Graduation Year, Surgeon Role. *P*-value is for the average yearly % change prior to 2020

	Mean (SD) of Logged Cases by Graduation Year											
Procedure	2012	2013	2014	2015	2016	2017	2018	2019	2020	Average Yearly % Change Prior to 2020 (95% CI)	*p*-value	% Change between 2019–2020
Cataract	152.8 (49)	157.7 (49)	163.7 (50)	175.8 (61)	183.6 (63)	188.4 (58)	198.1 (67)	208 (68)	162.2 (60)	**4.6 (4.3, 4.9)**	**< 0.001**	-22.0
Other Cataract	5.6 (6)	4.5 (4)	4.3 (5)	4.1 (6)	3.5 (4)	3.1 (4)	2.5 (3)	2.3 (3)	2.1 (8)	**-11.5 (-13.1, -9.9)**	**< 0.001**	-8.7
Total Laser	111.3 (77)	111.4 (79)	109.9 (75)	101.1 (72)	107.6 (80)	101.3 (69)	95.6 (56)	101.1 (63)	92.6 (57)	**-1.9 (-3.0, -0.8)**	**0.006**	-8.4
Total Cornea	12.4 (9)	12.0 (9)	12.2 (8)	12.0 (8)	12.6 (8)	12.3 (9)	12.2 (8)	12.7 (10)	11.6 (8)	0.4 (-0.4, 1.2)	0.278	-8.7
Keratorefractive	5.7 (12)	5.1 (14)	6 (16)	5.8 (15)	6.3 (17)	6.3 (17)	6.5 (15)	5.7 (12)	4.5 (11)	1.7 (-1.4, 4.9)	0.225	-21.1
Strabismus	26.1 (17)	26.9 (18)	24.7 (14)	25.2 (15)	24.6 (16)	23.7 (15)	23.3 (14)	23.5 (14)	22.9 (14)	**-1.9 (-2.7, -1.0)**	**0.002**	-2.6
Glaucoma	12.2 (7)	12.5 (7)	13.3 (7)	13.1 (8)	13.4 (8)	13.8 (9)	15.8 (12)	16.3 (13)	14.2 (11)	**4.0 (2.7, 5.4)**	**< 0.001**	-12.9
Retina Vitreous	6.5 (9)	6.1 (8)	5.8 (8)	5.9 (10)	5.7 (9)	6.1 (8)	5.5 (8)	6.4 (9)	5.4 (8)	-0.6 (-3.4, 2.2)	0.617	-15.6
Other Retinal	65.8 (68)	73.6 (67)	85.6 (82)	93.6 (102)	117.1 (118)	122.9 (134)	123.2 (121)	145.4 (138)	144.4 (142)	**11.9 (10.1, 13.7)**	**< 0.001**	-0.7
Oculoplastics	71.1 (36)	68.8 (35)	68.9 (32)	69.4 (35)	70 (37)	69.6 (39)	70.6 (39)	70.2 (37)	64.8 (35)	0.1 (-0.5, 0.7)	0.707	-7.7
Globe Trauma	10.2 (7)	9.4 (6)	8.9 (5)	8.9 (5)	9.4 (5)	9.2 (5)	9.1 (5)	9.6 (5)	9.0 (5)	-0.5 (-2.6, 1.6)	0.570	-6.3
Total	479.6 (170)	488.1 (165)	503.3 (179)	514.9 (200)	553.8 (228)	556.7 (233)	562.5 (208)	601.3 (245)	533.7 (230)	**3.2 (2.7, 3.8)**	**< 0.001**	-11.2

Similar patterns can be seen in the procedural categories for which ACGME requires a minimum number for graduation (Table 2). Average case logs in YAG capsulotomy, selective laser trabeculoplasty (SLT), filtering (glaucoma), and intravitreal injections had been increasing prior to 2020 (*p* < 0.05 for all) but decreased in 2020 (Fig. 1 C). In terms of absolute case volume, the numbers recorded in 2020 were comparable to recent years for cataract surgery (2014), YAG (2018), SLT (2018), keratoplasty (2013), other cornea (2018), glaucoma filtering (2017), glaucoma shunting (2013), retina vitreous (2018), intravitreal injections (2019), eyelid lacerations (2017), ptosis (2015), blepharoplasty (2016), and globe trauma (2018). However, for laser peripheral iridotomy (LPI), panretinal photocoagulation (PRP), pterygium, keratorefractive surgery, strabismus, total oculoplastics and orbit procedures, and chalazion, the 2020 case logs were the lowest reported numbers since 2012.

**Table 2:** Average Logged Cases across Graduating Residents by Graduation Year, Minimum Procedure Categories. *P*-value is for the average yearly % change prior to 2020. ^a^Minimum required is the surgeon and assistant role

	Mean (SD) of Logged Cases by Graduation Year											
Procedure	2012	2013	2014	2015	2016	2017	2018	2019	2020	Average Yearly % Change Prior to 2020 (95% CI)	*p*-value	% Change between 2019–2020
Cataract	152.8 (49)	157.7 (49)	163.7 (50)	175.8 (61)	183.6 (63)	188.4 (58)	198.1 (67)	208 (68)	162.2 (60)	**4.6 (4.3, 4.9)**	**< 0.001**	-22.0
YAG	16.5 (11)	16.5 (9)	18 (11)	18.3 (11)	20.6 (13)	20.9 (15)	22.2 (15)	23.5 (14)	22.8 (18)	**5.5 (4.7, 6.4)**	**< 0.001**	-3.0
SLT	12.3 (12)	11.8 (10)	13.1 (11)	13.1 (11)	15.5 (17)	15.6 (15)	16.1 (16)	16.5 (15)	16.1 (15)	**5.3 (3.7, 6.8)**	**< 0.001**	-2.4
LPI	15.2 (12)	15.2 (11)	15.8 (11)	14.8 (10)	15.8 (11)	16.1 (13)	15.4 (11)	15.6 (10)	13.6 (10)	0.4 (-0.1, 1.0)	0.113	-12.8
PRP	48.7 (55)	52.1 (66)	50 (62)	45.5 (59)	47.4 (65)	42.3 (51)	36.5 (36)	40.3 (47)	36.1 (39)	**-4.2 (-6.7, -1.5)**	**0.009**	-10.4
Keratoplasty^a^	9.9 (8)	9.2 (7)	10.4 (7)	10.4 (7)	11 (7)	10.5 (7)	10.5 (7)	10.1 (6)	9.5 (6)	1.1 (-0.8, 2.9)	0.212	-5.9
Pterygium	5.7 (7)	5.5 (6)	5.6 (6)	5.4 (5)	6 (6)	5.7 (6)	5.8 (6)	5.4 (5)	4.9 (5)	0.05 (-1.2, 1.3)	0.921	-9.3
Other Cornea	4.5 (4)	4.4 (4)	4.2 (4)	4 (4)	4 (4)	3.9 (3)	3.9 (4)	4.8 (6)	4.6 (4)	-0.4 (-4.3, 3.6)	0.794	-4.2
Keratorefractive^a^	13.8 (15)	15.4 (20)	15.2 (18)	14.3 (18)	16.2 (25)	16.2 (25)	16.4 (21)	14.1 (15)	11.9 (16)	0.9 (-2.2, 4.2)	0.498	-15.6
Strabismus	26.1 (17)	26.9 (18)	24.7 (14)	25.2 (15)	24.6 (16)	23.7 (15)	23.3 (14)	23.5 (14)	22.9 (14)	**-1.9 (-2.7, -1.0)**	**0.002**	-2.6
Glaucoma - Filtering	4.5 (4)	4.2 (4)	4.2 (4)	4.5 (5)	4.8 (4)	5.1 (5)	6.2 (7)	6 (7)	5.1 (6)	**5.6 (2.3, 9.1)**	**0.006**	-15.0
Glaucoma - Shunting	5.7 (4)	6 (4)	6.8 (5)	6.7 (5)	6.3 (5)	6.3 (5)	6.6 (5)	6.6 (5)	6.1 (5)	1.5 (-0.3, 3.3)	0.095	-7.6
Retina Vitreous^a^	29.9 (22)	31 (22)	30.1 (21)	28.9 (20)	29.3 (21)	28 (19)	26.2 (18)	26.6 (17)	26 (18)	**-2.2 (-3.3, -1.1)**	**0.003**	-2.3
Intravitreal Injections	64.7 (67)	72.6 (66)	85 (82)	93.1 (102)	116.7 (118)	122.6 (134)	122.8 (121)	145.1 (138)	144.1 (142)	**12.1 (10.3, 14.0)**	**< 0.001**	-0.7
Oculoplastics and Orbit	71.1 (36)	68.8 (35)	68.9 (32)	69.4 (35)	70 (37)	69.6 (39)	70.6 (39)	70.2 (37)	64.8 (35)	0.1 (-0.5, 0.7)	0.707	-7.7
Oculoplastics and Orbit - Eyelid Laceration	9.4 (7)	10.1 (8)	10.4 (7)	10.4 (8)	10.2 (7)	9.7 (7)	10.7 (8)	10.6 (8)	10.3 (7)	1.1 (-0.3, 2.4)	0.099	-2.8
Oculoplastics and Orbit - Chalazion	8.8 (7)	8.1 (6)	8 (5)	8.1 (5)	8.2 (5)	8.4 (6)	8.6 (7)	8.7 (7)	7.8 (5)	0.45 (-1.3, 2.3)	0.560	-10.3
Oculoplastics and Orbit - Ptosis	6.6 (8)	6.5 (7)	6.5 (7)	6.7 (6)	6.9 (7)	7.3 (7)	7.6 (7)	6.9 (6)	6.7 (7)	1.8 (0.0, 3.6)	0.054	-2.9
Oculoplastics and Orbit - Blepharoplasty	10.4 (10)	10.2 (10)	10.2 (9)	11.2 (10)	11.5 (10)	12.3 (11)	12.3 (12)	12.8 (12)	11.7 (11)	**3.6 (2.4, 4.8)**	**< 0.001**	-8.6
Globe Trauma	10.2 (7)	9.4 (6)	8.9 (5)	8.9 (5)	9.4 (5)	9.2 (5)	9.1 (5)	9.6 (5)	9.0 (5)	-0.5 (-2.6, 1.6)	0.570	-6.3

## Discussion

Overall, among graduating residents, reported procedural numbers across all categories decreased significantly in 2020. In an international survey conducted in early May 2020, the vast majority of ophthalmology residents and fellows reported a > 75% decrease in surgical training [8]. The deviation from expected trends in case logs as demonstrated in this study and the reports of severe disruption in surgical volume in the months prior to graduation suggest that the overall decreases of 11.2% and 9.6% in primary surgeon and S + A procedures, respectively, among graduating ophthalmology residents in 2020 were strongly influenced by COVID-19. High percentage decreases in average case logs were observed in both high volume (e.g. cataract) and low volume (e.g. keratorefractive surgery, retinal vitreous) procedures. The relative loss of dozens of cases for high volume procedures or 1–2 cases for rarer procedures may amount to valuable resident training experience lost. It is reassuring, however, that for certain procedures, the surgical numbers have not decreased below that of recent years.

Nonetheless, a critical limitation of using minimum surgical numbers to assess competency is that it is difficult to ascertain what threshold suffices as adequate experience, as the number required for competency may vary by trainee. Historically, programs have graduated residents who have had on average, lower case logs than residents who graduated during the COVID-19 pandemic. In fact, in 2020, the ACGME suspended minimum case log requirements and requested program directors evaluate competency for graduation [9]. Developing and implementing competency-based assessment to help adequately gauge resident preparation, as well as alternative training strategies (e.g. virtual reality simulators) [4–6] to provide residents with sufficient experience in a time with decreased surgical volume was a key task of residency program directors with the 2020 graduating classes [8].

The need for competency-based assessment and methods to supplement surgical training is not limited to the 2020 graduating class [9–11]. The surgical experience of upcoming graduating classes has been and continues to be impacted by the COVID-19 pandemic, but other interruptions in patient volume, such as loss of a high-volume surgeon or training site, can be equally disruptive to resident surgical and clinical education. This notion of shifting from minimum required numbers to competency-based requirements will likely continue to expand in graduate medical education, as studies have shown that residents achieve competency at different numbers [12]. While the ACGME has outlined levels of achievement of six competency milestones on a 1–5 scale (of which surgical skills falls under the Patient Care competency) [13], residency program directors have reported a need for more guidance and resources in implementing competency assessment even prior to the pandemic [14]. Our findings demonstrating the global decrease in surgical volume for residents during the pandemic only further emphasize that continued efforts to establish feasible, valid, and reliable assessment tools and surgical simulation for the variety of procedures that residents are expected to perform are needed [15]. Earlier, increased surgical exposure with the transition from traditional (one year of internship prior to three years of ophthalmology residency) to integrated four-year ophthalmology residency programs may also facilitate the establishment of competency-based assessment. Ultimately, with standardized tools widely incorporated into residency training, rigorous competency-based assessment can help identify areas of improvement for residents and allow for confidence in patient outcomes for residents deemed ready for independent practice [16]. In addition, competency-based assessment will allow for better evaluation of the effect of unexpected interruptions in surgical volume on surgical competency.

This study has several limitations. Most importantly, we are not able to assess any loss of competency due to the decreased volume of cases during the COVID-19 pandemic. In addition, while we present changes in resident case logs from 2019 to 2020, we are unable to attribute the decrease in resident case logs to COVID-19 disruptions alone. However, we were able to compare the changes experienced between 2019 and 2020 to the general trend in surgical volume in prior years. Additionally, the analysis is limited to case logs of graduating residents. While these senior residents may have had the most disruption in surgical volume in their final year, we cannot yet quantify changes to the surgical experience of more junior trainees during the pandemic. We also do not assess the operative experience of fellows, many of whom are in one-year programs. Further, while we used an official nationwide dataset, this data relies on accurate logging of cases by residents. Additionally, this study analyzed aggregated data, and thus, these results may not apply to the surgical experience of individual residents or residency programs. Finally, future work will need to evaluate the impact of lower surgical volume on resident surgical skills and patient outcomes.

## Conclusions

In conclusion, this study provides evidence that cases logged by residents greatly decreased during the time of COVID-19, however for many procedures with minimum requirements, the surgical numbers were not lower than that of recent years. The COVID-19 pandemic highlighted the vulnerability of ophthalmology residency programs to a significant interruption in surgical volume and may serve as an impetus for moving towards competency-based, rather than volume-based, assessment. Developing competency-based rather than volume-based requirements in graduate medical education may be valuable in assessing readiness for practice, particularly during periods with interruptions in surgical volume.

## Supplementary Information

**Additional file 1.**

## Data Availability

The datasets generated and/or analysed during the current study are available in the Accreditation Council for Graduate Medical Education repository, https://apps.acgme.org/ads/Public/Reports/Report/25.

## Abbreviations

COVID-19: coronavirus disease 2019
U.S.: United States
S + A: primary surgeon and surgical assistant (S + A)
ACGME: Accreditation Council for Graduate Medical Education
