# Supplementary material for: The impact of COVID-19 on ophthalmology resident surgical experience: a retrospective cross-sectional analysis
Source: BMC Med Educ. 2022 Mar 4;22:142. doi: 10.1186/s12909-022-03205-0 (PMC8894550; doi:10.1186/s12909-022-03205-0)
Supplement: Supplementary file 1 — Additional file 1. [file 12909_2022_3205_MOESM1_ESM.docx]

**Supplemental Table 1.** Average Logged Cases across Graduating Residents by Graduation Year, Surgeon and Assistant Role. P-value is for the average yearly % change prior to 2020.

|  | Mean (SD) of Logged Cases by Graduation Year | | | | | | | | |  |  |  |
| --- | --- | --- | --- | --- | --- | --- | --- | --- | --- | --- | --- | --- |
| Procedure | 2012 | 2013 | 2014 | 2015 | 2016 | 2017 | 2018 | 2019 | 2020 | Average Yearly % Change Prior to 2020 (95% CI) | p-value | % Change between 2019-2020 |
| Cataract | 231.4 (88) | 235.7 (84) | 238.5 (84) | 249.8 (91) | 259.5 (99) | 255.8 (91) | 263.6 (100) | 268.7 (93) | 219.1 (90) | **2.2 (2.0, 2.5)** | **<0.001** | **-18.5** |
| Other Cataract | 10.7 (12) | 9.4 (8) | 8.9 (9) | 8.3 (10) | 7.1 (7) | 6.4 (7) | 5.6 (12) | 4.6 (6) | 4.4 (12) | **-10.8 (-12.5, -9.1)** | **<0.001** | **-4.3** |
| Total Laser | 116.9 (78) | 117.6 (81) | 114.9 (76) | 106.4 (75) | 112 (82) | 105.2 (69) | 99.5 (56) | 104.7 (63) | 96.4 (57) | **-2.1 (-3.2, -1.1)** | **0.003** | **-7.9** |
| Total Cornea | 25.5 (14) | 24.3 (14) | 25.1 (13) | 24.8 (12) | 25.6 (13) | 24 (12) | 24 (12) | 23.9 (12) | 22.5 (11) | **-0.7 (-1.4, -0.1)** | **0.031** | **-5.9** |
| Keratorefractive | 13.8 (15) | 15.4 (20) | 15.2 (18) | 14.3 (18) | 16.2 (25) | 16.2 (25) | 16.4 (21) | 14.1 (15) | 11.9 (16) | 0.9 (-2.2, 4.2) | 0.498 | -15.6 |
| Strabismus | 42.1 (25) | 43.2 (29) | 39.8 (24) | 38.6 (25) | 36.3 (23) | 34.1 (23) | 33 (21) | 32.1 (19) | 32 (21) | **-4.4 (-5.4, -3.4)** | **<0.001** | **-0.3** |
| Glaucoma | 25.1 (14) | 25.3 (14) | 25.6 (15) | 24.9 (15) | 25.4 (16) | 23.6 (16) | 25.8 (18) | 25.6 (18) | 22.6 (16) | 0.01 (-0.9, 0.9) | 0.971 | -11.7 |
| Retina Vitreous | 29.9 (22) | 31 (22) | 30.1 (21) | 28.9 (20) | 29.3 (21) | 28 (19) | 26.2 (18) | 26.6 (17) | 26 (18) | **-2.2 (-3.3, -1.1)** | **0.003** | **-2.3** |
| Other Retinal | 69.5 (69) | 78.3 (68) | 89.4 (83) | 97.6 (104) | 120.6 (120) | 125.3 (134) | 125.5 (122) | 147.3 (138) | 146.6 (143) | **11.1 (9.4, 12.8)** | **<0.001** | **-0.5** |
| Oculoplastics | 119.2 (57) | 118.2 (69) | 114.5 (56) | 114.1 (62) | 113.3 (68) | 110.4 (64) | 111.6 (67) | 109.4 (59) | 102.4 (61) | **-1.2 (-1.5, -0.9)** | **<0.001** | **-6.4** |
| Globe Trauma | 13.9 (10) | 12.5 (8) | 11.3 (7) | 11 (7) | 11.2 (6) | 11 (6) | 10.7 (6) | 11.1 (6) | 10.4 (6) | **-2.8 (-5.3, -0.3)** | **0.033** | **-6.3** |
| Total | 698.1 (219) | 710.9 (222) | 713.4 (232) | 718.7 (251) | 756.6 (273) | 740 (265) | 741.9 (253) | 768 (269) | 694.4 (276) | **1.2 (0.9, 1.6)** | **<0.001** | **-9.6** |
